# A Survey of Perioperative and Postoperative Anesthetic Practices for Cesarean Delivery

**DOI:** 10.1155/2009/510642

**Published:** 2010-02-24

**Authors:** Leinani Aiono-Le Tagaloa, Alexander J. Butwick, Brendan Carvalho

**Affiliations:** Department of Anesthesia, Stanford University School of Medicine, 300 Pasteur Drive, Stanford, CA 94305, USA

## Abstract

The aim of this survey was to review cesarean delivery anesthetic practices. An online survey was sent to members of the Society of Obstetric Anesthesia and Perinatology (SOAP). The mode of anesthesia, preferred neuraxial local anesthetic and opioid agents, postoperative analgesic regimens, and monitoring modalities were assessed. 384 responses from 1,081 online survey requests were received (response rate = 36%). Spinal anesthesia is most commonly used for elective cesarean delivery (85% respondents), with 90% of these respondents preferring hyperbaric bupivacaine 0.75%. 79% used intrathecal fentanyl and 77% used morphine (median [range] dose 200 mcg [50–400]). 91% use respiratory rate, 61% use sedation scores, and 30% use pulse oximetry to monitor for postoperative respiratory depression after administration of neuraxial opioids. Postoperative analgesic regimens include: nonsteroidal anti-inflammatory agents, acetaminophen, oxycodone, and hydrocodone by 81%, 45%, 25%, and 27% respondents respectively. The majority of respondents use spinal anesthesia and neuraxial opioids for cesarean delivery anesthesia. There is marked variability in practices for monitoring respiratory depression postdelivery and for providing postoperative analgesia. These results may not be indicative of overall practice in the United States due to the select group of anesthesiologists surveyed and the low response rate.

## 1. Background

The cesarean delivery rate in the United States has been steadily increasing and accounts for approximately 31% of all births (>1 million cesarean deliveries per year) [1]. Changing patterns of obstetric management and an increase in maternal requests have both contributed to the increasing national rate of cesarean delivery. Important developments have also taken place in clinical obstetric anesthetic practice in recent years, including the introduction of new drugs (e.g., ropivacaine, extended-release epidural morphine [2]), equipment (e.g., small gauge pencil-point spinal needles), and analgesic techniques (e.g., patient-controlled epidural analgesia). However, surveys to specifically assess current anesthetic practices within the United States,and to determine the impact of these advances on Cesarean delivery anesthesia and analgesia are lacking [3–7]. An obstetric anesthesia workforce survey in the United States showed an increase in the rate of neuraxial anesthesia for patients undergoing Cesarean delivery, but no specific details about anesthetic techniques were described [6]. 

There is controversy regarding the extent of postoperative monitoring necessary to detect respiratory depression following the use of neuraxial opioids [8, 9]. The American Society of Anesthesiologists' (ASA) House of Delegates recently approved guidelines that address the prevention, detection, and management of respiratory depression associated with neuraxial opioid administration in all settings but did not specifically address obstetric patients [8, 10]. 

The aim of this study was to determine current obstetric anesthesia practices, post-operative analgesia practices and methods utilized to monitor for post-operative respiratory depression. 

## 2. Methods

Institutional review board approval was obtained with an exemption for consent. An online survey was created for this survey using http://www.freeonlinesurveys.com.  Permission was obtained from the Society for Obstetric Anesthesia and Perinatology (SOAP) to send an email with a link to the online survey to all anesthesiologists affiliated with the organization. Reminder emails were also sent to potential respondents if no reply was received two months after original notification. Responses were collected anonymously via the survey. 

The online survey consisted of 37 questions covering specific aspects of anesthetic practice related to cesarean delivery. The survey was initially reviewed by 10 experienced anesthesiologists, who regularly participate in obstetric anesthesia care, at the investigators' institution. Feedback from these anesthesiologists was used to refine the survey to ensure accuracy, validity, and reliability before being submitted to SOAP members. 

The survey questions were designed to obtain basic demographic information about the anesthesiologist's hospital including hospital type (private, county, military, or teaching), number of deliveries per annum, rate of cesarean delivery, and anesthesia coverage (dedicated 24 hours in-house obstetric anesthesia coverage, shared coverage between the operating rooms, and delivery suite, or other variations of coverage options). Information was also collected regarding the anesthesiologist's own involvement in the provision of obstetric anesthesia services: daytime, on-call only, or a combination of both; the average number of days worked per week. Additional questions were structured to determine the anesthesiologist's preferred anesthetic technique for patients requiring either elective or urgent cesarean deliveries including specific information of neuraxial drugs (local anesthetics and adjuncts); modes of postoperative analgesia, and the extent and duration of postoperative monitoring after cesarean delivery following neuraxial opioid administration.

### 2.1. Statistical Analysis

Statistical analysis was conducted using Microsoft Excel and SPSS statistical package Version 11 (Chicago, IL). Descriptive statistics were used to summarize demographic and practice data. Data are expressed as mean ± SD, median (IQR), and number (percentage) as indicated. Normal distribution was determined using QQ plots and the Kolmogorov-Smirnov test.

## 3. Results

**Figure 1:**
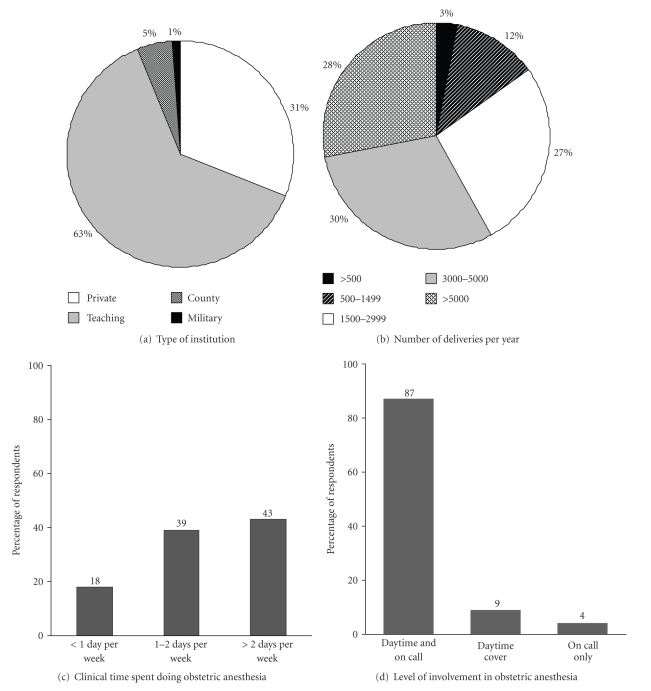
The Demographics of Survey Respondents' Clinical Practice.

The online survey was emailed to 1,081 SOAP members' email addresses and 384 responses were received (response rate = 36%). The demographics of respondents' clinical practice are shown in Figure 1. The median (IQR) reported cesarean delivery rate at hospitals where respondents were employed was 30% (27–35%). During daytime hours, 75% of respondents provide dedicated in-house obstetric anesthesia coverage, 17% provide in-house obstetric anesthesia care shared with the main operating room, 2% provide offsite coverage, and 6% specified a variety of other arrangements to cover the obstetric service. During nighttime hours, 63% of respondents provide dedicated in-house obstetric anesthesia coverage, 29% provide in-house obstetric anesthesia care shared with the main operating room, 6% provide offsite coverage, and 2% specified a variety of other arrangements to cover the obstetric service during nighttime hours. 

### 3.1. Elective Cesarean Delivery

**Figure 2:**
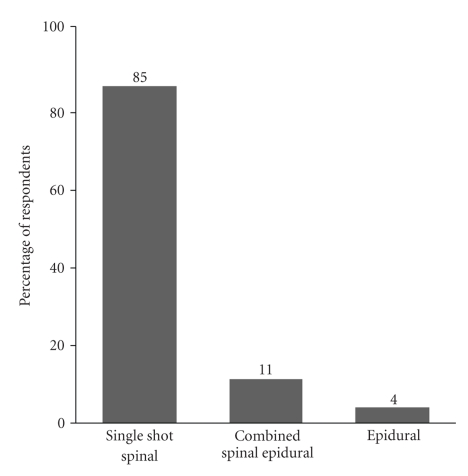
Preferred Regional Technique for Elective Cesarean Delivery.

All respondents stated that a neuraxial anesthetic technique is routinely used for elective cesarean delivery at their affiliated institution. The preferred neuraxial anesthetic techniques for elective cesarean delivery are outlined in Figure 2. Pencil point spinal needles (Whitacre, Pencan, and Sprotte needles) were most commonly used (94% of respondents) for administering spinal anesthesia; only 5% of respondents stated that a cutting (Quincke) spinal needle is routinely used. Sixty three percent use a 25-gauge needle, 16% a 27-gauge, 13% a 24-gauge, and 8% used other needle sizes for administering single shot spinal anesthesia. 

The loss of resistance technique for epidural placement varied among respondents (43% using saline; 42% using air; 13% using saline/air combination; 2%, using local anesthetic agents). 

Ninety percent of respondents preferred hyperbaric bupivacaine 0.75% for spinal anesthesia (median dose (range) = 12 mg (6–15 mg)). Hypobaric bupivacaine 0.5% and ropivacaine were less commonly used by respondents (8% and 2%, resp.). Fifty-four percent of respondents used a combination of fentanyl, and morphine, 77% used only fentanyl and 79% used only morphine. The median (range) dose of morphine was 200 mcg (50–400 mcg). Other routinely administered spinal adjuvants included diamorphine (2%), sufentanil (2%), and epinephrine (4%).

### 3.2. Urgent Cesarean Delivery

The preferred choice of local anesthetic to convert a preexisting labor epidural for urgent cesarean delivery was 2% lidocaine (74% respondents) followed by chloroprocaine (21%); a minority of respondents (5%) use bupivacaine, ropivacaine, or combination regimens. The median (range) volume of 2% lidocaine commonly used to convert a labor epidural to regional anesthesia was 20 mL (10–25 mL). The most commonly used epidural adjuncts are outlined in Table 1.

### 3.3. Postoperative Monitoring for Respiratory Depression

**Figure 3:**
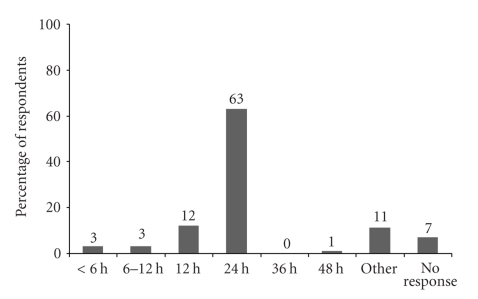
Duration of Post-Cesarean Delivery Monitoring for Respiratory Depression.

**Figure 4:**
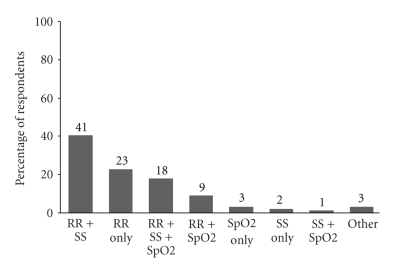
Types of Monitoring Used to Detect Respiratory Depression. RR = respiratory rate, SS = sedation score, SpO2 = oxygen pulse oximetry.

The majority of respondents (93%) reported that their institution had a protocol for monitoring patients after administration of neuraxial opioids. The duration of post-cesarean delivery monitoring among the respondents is outlined in Figure 3. The modalities (respiratory rate, sedation score, pulse oximetry) used routinely to detect respiratory depression are shown in Figure 4. One respondent utilized capnography with respiratory rate and sedation monitoring for transferring patients to the recovery room following surgery.

### 3.4. Postoperative Analgesia

Seventy-nine percent of respondents removed the epidural after surgery; 21% used the epidural for post-cesarean epidural analgesia. Of respondents that used post-cesarean epidural analgesia, nearly half (48%) used patient-controlled epidural analgesia (PCEA) with a continuous background infusion, with the remainder preferring an intermittent bolus technique (24%), continuous epidural infusion (21%), or PCEA alone (7%). 

Only 12% of all respondents routinely use intravenous patient-controlled analgesia (IV PCA), with morphine being the most popular agent. Nonsteroidal anti-inflammatory agents (NSAIDs) were used by most respondents (81%), with 42% using “around-the-clock” dosing, 51% administering NSAIDS on a PRN basis, and 7% preferring other dosing regimens (most commonly a single dose of NSAID in the recovery room post-cesarean delivery). 

Acetaminophen was the most commonly used oral analgesic following cesarean delivery (45% respondents). Twenty-five percent used oxycodone, 27% used hydrocodone, 12% used codeine, and 6% used tramadol routinely. Twenty one percent of respondents stated that postoperative analgesia was managed directly by the obstetric team.

## 4. Discussion

The widespread use of neuraxial anesthesia for patients undergoing routine cesarean delivery is confirmed by our survey. Our results are supported by similar high rates of neuraxial anesthesia reported in previous surveys of obstetric anesthesia practice [6, 7]. 

Spinal anesthesia was the most popular choice for elective cesarean delivery among respondents in our survey. A United Kingdom survey in 2002 reported a similar rate of spinal anesthesia (87% anesthesiologists) for elective cesarean delivery [7]. Spinal anesthesia has been shown to be more cost effective, less technically challenging, and can achieve adequate surgical anesthesia in a shorter time-frame as compared with epidural anesthesia [11]. Although there are reports of improved intraoperative analgesia with spinal compared to epidural anesthesia, a meta-analysis of ten studies involving 751 women found no differences between spinal and epidural techniques with regard to failure rate, additional requests for intraoperative analgesia, conversion to general anesthesia, maternal satisfaction, postoperative analgesia requirements, and neonatal outcomes [12]. 

The vast majority of respondents in our survey use small gauge, pencil-point spinal needles for performing spinal anesthesia. This practice is substantiated by a lower incidence of post-dural puncture headache with pencil point needles compared to cutting needles (2.7% versus 0.4% with 27-gauge Whitacre needle versus 27-gauge Quincke needle, resp. [13]). Most anesthesiologists (63%) in our survey preferred using 25-gauge spinal needles, which may be due to a more reliable “pop” sign (as an endpoint for breaching the dura) compared with smaller 27-gauge needles [14]. 

The use of a combined-spinal epidural (CSE) technique offers rapid onset and effective anesthesia, and the advantage of an epidural catheter for extending the duration of regional anesthesia or for managing intraoperative breakthrough pain. In our survey, only 11% of anesthesiologists reported routinely using CSE for routine cesarean delivery, which is consistent with a previous survey in the United Kingdom that reported a similar low percentage of anesthesiologists using a CSE technique (7%) [7]. Despite the potential advantages associated with a CSE technique, the low observed rates may be due to increased cost, longer block insertion time, and the lack of need for prolonged anesthesia. 

Epidural anesthesia is characteristically performed using a loss-of-resistance technique during needle placement. Loss-of-resistance technique using saline (as opposed to air) is associated with improved analgesia and less breakthrough pain, a lower incidence of post-dural puncture headaches, and fewer complications [15–17]. Despite these advantages, respondents in our survey were divided between the two techniques (43% prefer saline, 42% prefer air, and 13% prefer a saline/air combined technique). A previous survey of members of the Obstetric Anaesthetists' Association in the United Kingdom reported that 37% and 53% respondents use loss-of-resistance to air or saline, respectively, [18]. It is likely that the similar rates for loss-of-resistance using air or saline may be due to a number of factors including: obstetric anesthesia training and experience with only one technique, and the perceived difficulty to determine whether an accidental dural puncture has occurred with the use of saline [15]. In addition, there may be concern in visually confirming the presence of cerebrospinal fluid using a loss of resistance technique with saline following dural puncture. However saline and cerebrospinal fluid can be differentiated by simple bedside testing (temperature-using the back of the gloved hand, or pH, glucose and protein using urine testing sticks) [19]. It is interesting that 13% of respondents used a saline/air combination; the advantages of this combination technique are not described in the literature. The use of local anesthetic (2% of respondents) for the loss of resistance technique should be strongly discouraged because of a potential risk of accidental administration of local anesthetic into the intrathecal or intravascular space. 

The majority of respondents (90%) in this survey use hyperbaric bupivacaine for spinal anesthesia, and the use of lidocaine and ropivacaine as alternatives were less popular. The reported median dose of intrathecal bupivacaine was 12 mg, which is consistent with the results from a previous dose-finding study for patients undergoing cesarean delivery (ED 95 = 11 mg) [20]. Doses of intrathecal bupivacaine ≤10 mg have been proposed to reduce hypotension and speed up recovery; however, reliable anesthesia of adequate duration cannot be assured for cesarean delivery [21, 22]. Current evidence supports the use of hyperbaric (as compared with hypobaric) bupivacaine due to increased initial block height consistency, improved anesthetic reliability, and less hypotension due to slower block onset [23, 24].

Most respondents reported using intrathecal opioid adjuvants for spinal anesthesia for cesarean delivery. These results suggest that anesthesiologists in our survey are aware of the advantages of using lipophilic opioids such as fentanyl (to optimize intraoperative analgesia) [25, 26], and hydrophilic opioids such as morphine (for providing an adequate duration of effective post-cesarean analgesia) [27, 28]. The median dose of intrathecal morphine reported was 200 mcg which is the upper end of an apparent analgesic ceiling (approximately 50–200 mcg); larger doses may increase side effects without providing additional analgesic benefit [27]. 

In our survey, lidocaine is the preferred local anesthetic (used by 79% respondents) for conversion to regional anesthesia for urgent cesarean delivery in patients with a preexisting labor epidural. These results differ from a recent postal survey assessing obstetric anesthetic practice in the United Kingdom, which reported that bupivacaine 0.5% was the most commonly used agent (used as the sole agent or in combination with other local anesthetics) [29]. Lidocaine has a lower potential for cardiotoxicity in the event of local anesthetic toxicity. In addition, when extending epidural analgesia for cesarean delivery, epidural lidocaine-bicarbonate-epinephrine halves the block onset time (7 versus 14 minutes to attain loss to touch at T5) when compared with levobupivacaine [30]. 

Respondents in our survey reported a low rate (21%) of epidural analgesia following cesarean delivery. It is possible that the limitations on maternal mobility, increased costs (nurse training, equipment, epidural medications), catheter dislodgement, and the potential for catheter-related complications (e.g., hematoma, infection) account for the low rate of post-operative epidural analgesia compared to single-bolus doses of intrathecal or epidural morphine. Most respondents in our survey (81%) prescribed NSAIDs for postoperative analgesia following cesarean delivery. This practice is consistent with evidence from clinical studies confirming that NSAIDs greatly enhance the analgesic efficacy of intrathecal morphine in patients following cesarean delivery [31, 32]. 

Our survey reflects marked variability in respondents' preferred practices for monitoring respiratory depression following cesarean delivery. Modes of assessment included respiratory rate, sedation score, and/or pulse oximetry. The ASA Task Force guidelines are general guidelines that include but do not specifically address the method and duration of monitoring obstetric patients post-Cesarean delivery [8, 10]. The reported duration of monitoring for respiratory depression was also variable; however, we did not assess whether this was related to the choice of intrathecal opioid for neuraxial anesthesia. The majority (77%) of respondents in our survey use intrathecal morphine, and the ASA Task Force recommends that respiratory monitoring after neuraxial morphine should occur at least every hour for the first 12 hours, then every 2 hours for the next 12 hours [10]. 

We acknowledge that there are a number of limitations to our study. We chose to survey members of the SOAP and a selection bias towards a subgroup of anesthesia providers familiar with current obstetric anesthesia practices is likely to be present. In addition, two thirds of respondents came from academic teaching hospitals. As a consequence, our results may not be representative of general obstetric anesthesia practice or be indicative of overall practice within the United States. A subgroup with an interest in obstetrics potentially represents the “best” obstetric anesthesia practice. This makes the wide differences and lack of consensus in anesthetic practice, especially with regard to post-operative monitoring and analgesic regimens, even more striking. Despite the use of email and an online website to target our study population, the response rate to our survey was lower than expected (36%). Target response rates of 70 to 80% are preferable in order to infer meaningful results from survey data [33]. However a, recent published postal survey of obstetric anesthesia workforces similarly reported a low response rate (29%) [6], and there have been suggestions that multiple requests for surveys result in lower response rates [34]. We were unable to individually contact nonresponders, as individual email addresses were not provided by SOAP, which may have resulted in responder bias. It is possible that answers from survey respondents may have been different from nonrespondents, resulting in a biased estimate of the characteristics of the population. However, our survey responses have face validity, and our results are meaningful in demonstrating common clinical practice preferences as well as variability among practitioners. 

In conclusion, the majority of respondents in our survey of SOAP members use spinal anesthesia with intrathecal bupivacaine, fentanyl, and morphine for uncomplicated elective cesarean delivery. Lidocaine is the preferred local anesthesia to convert a preexisting labor epidural to regional anesthesia for urgent cesarean delivery. Based on our results, there is marked variability in practices for monitoring respiratory depression following cesarean delivery and for providing postoperative analgesia among survey respondents. Due to selection bias of surveying a subgroup of anesthesiologists with an interest in obstetric anesthesia and the low response rate, these findings may not indicative of overall practice within the United States. Further studies are needed to explore the basis for the reported variations in obstetric anesthetic practices. 

## Figures and Tables

**Table 1:** Agents added to epidural local anesthetic solution for urgent cesarean delivery.

Agent	Number (percentage) of respondents
Fentanyl	204 (54)
Morphine	143 (37)
Sodium bicarbonate	162 (42)
Epinephrine	129 (34)
Sufentanil	7 (2)

## List of Abbreviations

SOAP: Society of Obstetric Anesthesia and Perinatology
PCEA: Patient-controlled epidural analgesia
CSE: Combined-spinal epidural
ASA: American Society of Anesthesiologists
IV PCA: Intravenous patient controlled analgesia
NSAIDs: Nonsteroidal anti-inflammatory agents.

## References

[1] Martin JA, Hamilton BE, Sutton PD (2007). Births: final data for 2005. *National Vital Statistics Reports*.

[2] Carvalho B, Roland LM, Chu LF, Campitelli VA, Riley ET (2007). Single-dose, extended-release epidural morphine (DepoDur) compared to conventional epidural morphine for post-Cesarean pain. *Anesthesia and Analgesia*.

[3] Stamer UM, Wiese R, Stuber F, Wulf H, Meuser T (2005). Change in anaesthetic practice for Caesarean section in Germany. *Acta Anaesthesiologica Scandinavica*.

[4] Van Houwe P, Heytens L, Vercruysse P (2006). A survey of obstetric an anaesthesia practice in Flanders. *Acta Anaesthesiologica Belgica*.

[5] Chan YK, Ng KP (2000). A survey of the current practice of obstetric anaesthesia and analgesia in Malaysia. *Journal of Obstetrics and Gynaecology Research*.

[6] Bucklin BA, Hawkins JL, Anderson JR, Ullrich FA (2005). Obstetric anesthesia workforce survey: twenty-year update. *Anesthesiology*.

[7] Jenkins JG, Khan MM (2003). Anaesthesia for caesarean section: a survey in a UK region from 1992 to 2002. *Anaesthesia*.

[8] Carvalho B (2008). Respiratory depression after neuraxial opioids in the obstetric setting. *Anesthesia and Analgesia*.

[9] Shapiro A, Zohar E, Zaslansky R, Hoppenstein D, Shabat S, Fredman B (2005). The frequency and timing of respiratory depression in 1524 postoperative patients treated with systemic or neuraxial morphine. *Journal of Clinical Anesthesia*.

[10] Horlocker TT, Burton AW, Connis RT (2009). Practice guidelines for the prevention, detection, and management of respiratory depression associated with neuraxial opioid administration. *Anesthesiology*.

[11] Riley ET, Cohen SE, Macario A, Desai JB, Ratner EF (1995). Spinal versus epidural anesthesia for cesarean section: a comparison of time efficiency, costs, charges, and complications. *Anesthesia and Analgesia*.

[12] Ng K, Parsons J, Cyna AM, Middleton P (2004). Spinal versus epidural anaesthesia for caesarean section. *Cochrane Database of Systematic Reviews*.

[13] Santanen U, Rautoma P, Luurila H, Erkola O, Pere P (2004). Comparison of 27-gauge (0.41-mm) Whitacre and Quincke spinal needles with respect to post-dural puncture headache and non-dural puncture headache. *Acta Anaesthesiologica Scandinavica*.

[14] Kathirgamanathan A, Hawkins N (2007). Reliability of the ‘pop’ sign as an indicator of dural puncture during obstetric spinal anaesthesia: a prospective observational clinical study. *Anaesthesia*.

[15] Shenouda PE, Cunningham BJ (2003). Assessing the superiority of saline versus air for use in the epidural loss of resistance technique: a literature review. *Regional Anesthesia and Pain Medicine*.

[16] Leo S, Lim Y, Sia ATH (2008). Analgesic efficacy using loss of resistance to air vs. saline in combined spinal epidural technique for labour analgesia. *Anaesthesia and Intensive Care*.

[17] Van de Velde M (2006). Identification of the epidural space: stop using the loss of resistance to air technique!. *Acta Anaesthesiologica Belgica*.

[18] Howell TK, Prosser DP, Harmer M (1998). A change in resistance? A survey of epidural practice amongst obstetric anaesthetists. *Anaesthesia*.

[19] El-Behesy BAZ, James D, Koh KF, Hirsch N, Yentis SM (1996). Distinguishing cerebrospinal fluid from saline used to identify the extradural space. *British Journal of Anaesthesia*.

[20] Ginosar Y, Mirikatani E, Drover DR, Cohen SE, Riley ET (2004). ED50 and ED95 of intrathecal hyperbaric bupivacaine coadministered with opioids for Cesarean delivery. *Anesthesiology*.

[21] Ben-David B, Miller G, Gavriel R, Gurevitch A (2000). Low-dose bupivacaine-fentanyl spinal anesthesia for cesarean delivery. *Regional Anesthesia and Pain Medicine*.

[22] Bryson GL, MacNeil R, Jeyaraj LM, Rosaeg OP (2007). Small dose spinal bupivacaine for Cesarean delivery does not reduce hypotension but accelerates motor recovery. *Canadian Journal of Anesthesia*.

[23] Vercauteren MP, Coppejans HC, Hoffmann VL, Saldien V, Adriaensen HA (1998). Small-dose hyperbaric versus plain bupivacaine during spinal anesthesia for cesarean section. *Anesthesia and Analgesia*.

[24] Carvalho B, Durbin M, Drover DR, Cohen SE, Ginosar Y, Riley ET (2005). The ED50 and ED95 of intrathecal isobaric bupivacaine with opioids for cesarean delivery. *Anesthesiology*.

[25] Palmer CM, Voulgaropoulos D, Alves D (1995). Subarachnoid fentanyl augments lidocaine spinal anesthesia for cesarean delivery. *Regional Anesthesia*.

[26] Dahlgren G, Hultstrand C, Jakobsson J, Norman M, Eriksson EW, Martin H (1997). Intrathecal sufentanil, fentanyl, or placebo added to bupivacaine for cesarean section. *Anesthesia and Analgesia*.

[27] Dahl JB, Jeppesen IS, Jorgensen H, Wetterslev J, Moiniche S (1999). Intraoperative and postoperative analgesic efficacy and adverse effects of intrathecal opioids in patients undergoing cesarean section with spinal anesthesia: a qualitative and quantitative systematic review of randomized controlled trials. *Anesthesiology*.

[28] Pan PH (2006). Post cesarean delivery pain management: multimodal approach. *International Journal of Obstetric Anesthesia*.

[29] Regan KJ, O’Sullivan G (2008). The extension of epidural blockade for emergency Caesarean section: a survey of current UK practice. *Anaesthesia*.

[30] Allam J, Malhotra S, Hemingway C, Yentis SM (2008). Epidural lidocaine-bicarbonate-adrenaline vs levobupivacaine for emergency Caesarean section: a randomised controlled trial. *Anaesthesia*.

[31] Angle PJ, Halpern SH, Leighton BL, Szalai JP, Gnanendran K, Kronberg JE (2002). A randomized controlled trial examining the effect of naproxen on analgesia during the second day after cesarean delivery. *Anesthesia and Analgesia*.

[32] Cardoso MMSC, Carvalho JCA, Amaro AR, Prado AA, Cappelli EL (1998). Small doses of intrathecal morphine combined with systemic diclofenac for postoperative pain control after cesarean delivery. *Anesthesia and Analgesia*.

[33] Bruce J, Chambers WA (2002). Questionnaire surveys. *Anaesthesia*.

[34] Sheehan K (2001). Email survey response rates: a review. *Journal of Computer Mediated Communication*.

